# 3D-printed titanium porous prosthesis combined with the Masquelet technique for the management of large femoral bone defect caused by osteomyelitis

**DOI:** 10.1186/s12891-024-07576-x

**Published:** 2024-06-17

**Authors:** Zhuo Chen, Yong Xing, Xingcai Li, Bingchuan Liu, Ning Liu, Yaping Huo, Yun Tian

**Affiliations:** 1 Department of Orthopedics, Peking University Third Hospital, No.49, North Garden Rd, HaiDian District, Beijing, 100191 China; 2 Beijing AK Medical Co., Ltd, Changping District, Beijing, China

**Keywords:** Infected bone defect, Osteomyelitis, Femoral defects, Three-dimensional printed prosthesis, Defect reconstruction

## Abstract

**Background:**

The treatment of infected bone defects remains a clinical challenge. With the development of three-dimensional printing technology, three-dimensional printed implants have been used for defect reconstruction. The aim of this study was to investigate the clinical outcomes of three-dimensional printed porous prosthesis in the treatment of femoral defects caused by osteomyelitis.

**Methods:**

Eleven patients with femoral bone defects following osteomyelitis who were treated with 3D-printed porous prosthesis at our institution between May 2017 and July 2021, were included. Eight patients were diagnosed with critical-sized defects, and the other three patients were diagnosed with shape-structural defects. A two-stage procedure was performed for all patients, and the infection was eradicated and bone defects were occupied by polymethylmethacrylate spacer during the first stage. The 3D-printed prosthesis was designed and used for the reconstruction of femoral defects in the second stage. Position of the reconstructed prostheses and bone growth were measured using radiography. The union rate, complications, and functional outcomes at the final follow-up were assessed.

**Results:**

The mean length of the bone defect was 14.0 cm, union was achieved in 10 (91%) patients. All patients showed good functional performance at the most recent follow-up. In the critical-sized defect group, one patient developed a deep infection that required additional procedures. Two patients had prosthetic dislocations. Radiography demonstrated good osseous integration of the implant–bone interface in 10 patients.

**Conclusion:**

The 3D printed prostheses enable rapid anatomical and mechanically stable reconstruction of extreme femur bone defects, effectively shortens treatment time, and achieves satisfactory clinical outcomes.

## Introduction

The treatment of infected bone defects remains a clinical challenge, especially for large and irregular defects. Various methods, such as the vascularized fibula graft [1], and the Masquelet-induced membrane [2–4], Ilizarvo bone transport [5–7], and structural allograft techniques [8], have been reported for the treatment of large bone defects with good clinical results. However, for defects large in length, the traditional technique requires a longer treatment period and is associated with various complications, such as pin tract infections, docking site nonunion, and joint stiffness. Reducing the treatment cycle and incidence of complications, obtaining good mechanical stability, and improving the fusion rate have become key to successful treatment [9].

With the development of three-dimensional (3D) printing technology, 3D-printed implants have been used for defect reconstruction. They have several advantages, such as their lack of need for additional bone grafting, immediate stability, customized matched shape to fill the defect, and ability to accurately correct length discrepancies, which make them widely used in the treatment of bone defects caused by tumor resection [10–15], trauma [16] and infection [17, 18], especially for large extremity defects. However, some researchers have reported a high incidence of complications during long-term follow-up, which significantly affect the reconstruction survival rate. In a recent study [16–19], the porous scaffold structure enable not only short-term effect but also long-term stability. By adjusting the porosity and pore size to fit the elastic modulus of the bone, stress shielding effects and mechanical failure can be effectively prevented [20–22], offering the possibility of long-term stability and good conditions for osseointegration. In clinical practice, this porous prosthesis has been used for the reconstruction of bone defects after tumor resection of the extremity diaphysis, and the pelvis has demonstrated good short-term bone ingrowth effects [23–26]. Thus, to shorten the treatment cycle of the infected bone defect and compensate for the insufficiency of conventional techniques, we used a 3D-printed porous prosthesis in the treatment of femoral defects caused by osteomyelitis. We have used an intramedullary nail to realize micromotion with certain parameters, while maintaining prosthesis stability. In our previous research, short-term effectiveness was demonstrated both in animals and clinics [22, 27, 28]. In the present study, we prospectively evaluated a group of 11 patients with femoral bone defects who underwent reconstruction with a 3D-printed porous prosthesis, with a mean follow-up period of 43 months. The aim was to investigate the long-term stability of this novel fixation system, its complication rate, and the effects of osseointegration.

## Materials and methods

### Patients

We have prospectively studied 11 consecutive patients (7 male and 4 female) with femoral bone defects caused by osteomyelitis or infected nonunion after fracture who were treated at our institution between May 2017 and July 2021. Patients with defects due to tumor resection or Charcot arthropathy and those with less than 1 year of follow-up were excluded. The mean patient age was 52.4 years (range: 24–79 years). The patients underwent a mean of 4.4 debridements before prosthetic implantation. The mean length of the bone defect was 14.0 cm, and the largest defect was 26.1 cm. The mean follow-up was 43.0 months (range: 24–68 months). All patients provided informed consent for their participation.

### Surgical technique

Similar to the Masquelet technique [3], we used a two-stage treatment approach. In the first stage, the patients were placed on an orthopedic operating table. A lateral incision was made to clean up the infected soft tissue and necrotic bone until bleeding was observed at the bone margin. The proximal and distal interfaces were trimmed to a plane perpendicular to the long axis of the femoral diaphysis. After reaming the femoral canal, the entire surgical area was repeatedly flushed with hydrogen peroxide and iodophor solution to ensure that the local infection was under control. The bone tissue, surrounding soft tissue, and irrigation fluid were retained for bacterial culture during surgery. A vancomycin-loaded polymethylmethacrylate (PMMA) spacer which consisted of 40 g PMMA bone cement and 2 g vancomycin was used to fill the spaces and canals. According to the length and diameter of the defects, the PMMA should be slightly larger to ensure sufficient space for the prosthesis and maintain the length of the lower limbs. Vacuum sealing drainage (VSD) was used for continuous wound irrigation, and the drainage fluid was retained for 3 days postoperatively for bacterial culture. Debridement was performed weekly until all antibiograms and cultures were negative. PMMA spacing should be performed for 6–8 weeks to generate a high-quality induction membrane and confirm that no infection remains. External fixation was used to maintain stability of the extremity in nine patients with critical-size defects. After the first stage of treatment, patients were asked to undergo computed tomography (CT) of both lower extremities, which was used to design and fabricate the 3D-printed prosthesis during the waiting period. Based on CT, patients with radiographically apparent bone gaps were defined as having critical defects. Patients with continuous bone cortex were defined as having shape-structural defects.

Before the second stage of treatment, the soft tissue and wound conditions, white blood cell count levels (WBC), leukocyte counts, C-reactive protein levels (C-rp), and erythrocyte sedimentation rates (ESR) were evaluated to ensure local infection control. The PMMA spacer was removed by carefully incising the membrane. The 3D-printed prostheses of all patients were inserted with 2 g vancomycin into the drug-loaded hollows. For patients with critical defects, the intramedullary nails (Smith & Nephew Medical Ltd.) were used to keep the prosthesis stable while distributing stress on the prosthesis. The wound was closed in layers, as usual. Postoperatively, according to pathogen susceptibility tests, intravenous and oral empiric antibiotics were administered for 1 month. During the waiting period of the first and second stages, all patients were required to take oral second-generation cephalosporins.

### Prosthesis design

**Fig. 1:**
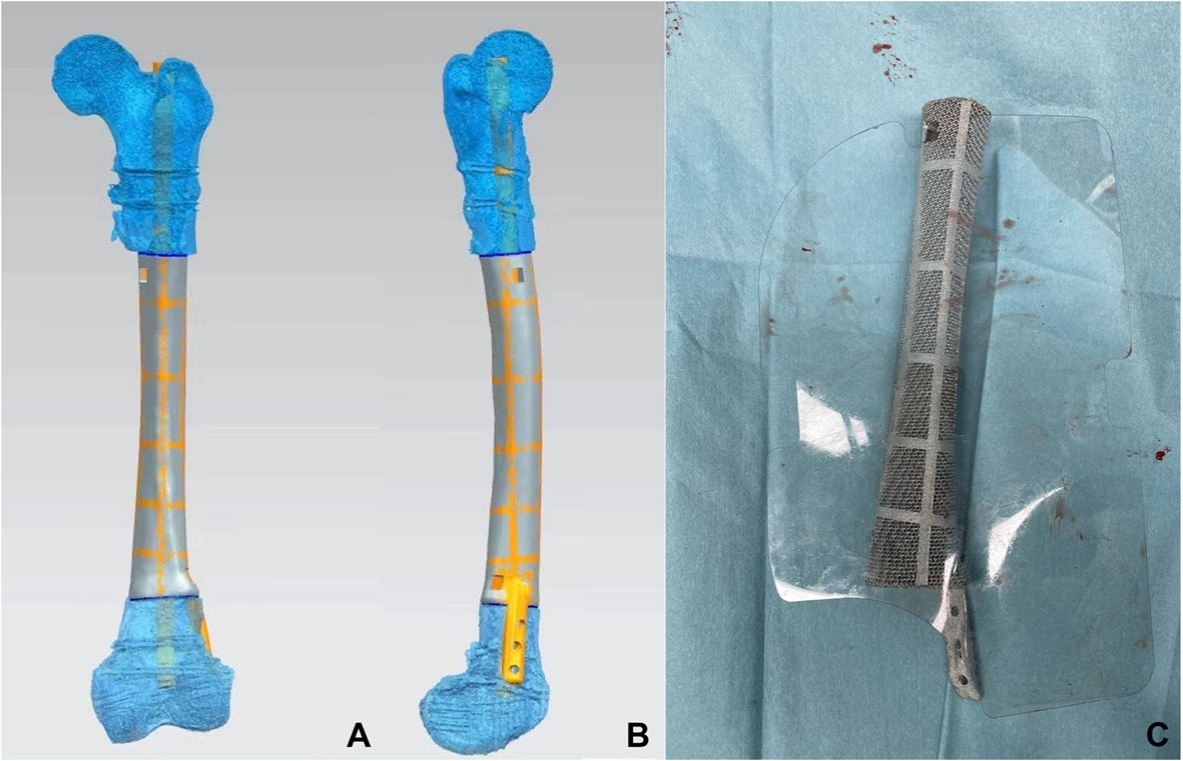
**A** and **B** the design drawings of the 3D printed prosthesis. **C** the finished photo of 3D printed prosthesis, a lateral plate was added to ensure strength

The prosthesis design process was conducted between the first and second stages with the participation of AK Medical Holding Ltd. According to the workflow [29], the 3D structure was first projected using the Mimics software (Mimics 21.0; Materialise, Leuven, Belgium) based on the length of the healthy femur and anatomical shape of the defect area. The size of the prosthesis interface should be smaller in diameter than that of the host bone to help new bone tissue to crawl and grow towards the prosthesis. The material of the prosthesis was titanium alloy (Ti6Al4V), according to previous literature and our animal experiments [20–22]. The prosthesis was constitutive of regular icosahedron units with a porosity of approximately 60–70% and a pore size of 625 ± 70 μm. All prostheses used in critical-size defects had a hollow design to mimic the structure of the medullary cavity (Fig. 1). To facilitate the application of intramedullary nails, the diameter of the hollow portion was designed 1–1.5 mm larger than the diameter of the intramedullary nail. According to our previous finite element analysis based on ABAQUS 6.13, the stress was mostly concentrated at the prosthesis interface [30]; therefore, the intramedullary nail could effectively disperse the lateral stress on the prosthesis while limiting the lateral dislocation of the prosthesis. By this means, the risk of aseptic loosening and prosthetic fracture was reduced, which aids the immediate stabilization of the prosthesis. In addition, the presence of an intramedullary nail allowed for micromotion between the prosthesis and bone interface to facilitate osseointegration. Three locking nails were used for distal femur to prevent prosthesis subsidence.

When the defect involved the metaphysis, proper and rigid fixation was difficult to achieve because of its special anatomical structure and insufficient anchorage length [25, 31]. Hence, the prosthesis was designed with a lateral wing plate that was 3–4 cm long and fixed with three to four screws to enhance its stability. Although bone growth is affected to some extent by the stress shielding effect [22, 30], it effectively prevents prosthesis failure due to excessive micromotion.

If the distal bone of the metaphysis was considered insufficient to lock the intramedullary nail, an integrated intramedullary nail prosthesis was applied. The distal portion of the prosthesis was stabilized using four screws that penetrated the prosthesis to fix the distal bone.

**Fig. 2:**
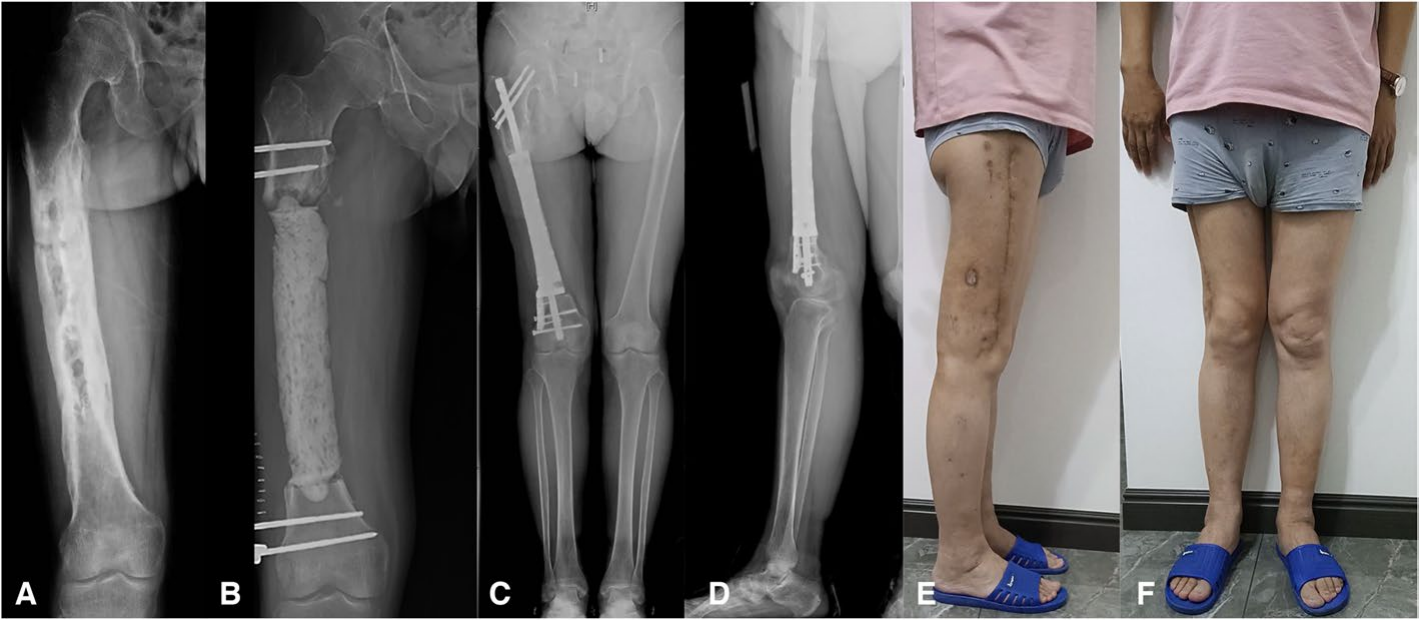
**A** A 51-year-old patient suffered osteomyelitis after a femur injury. **B** After debridement three times, an external fixator was applied, and PMMA was used to fill the spacer. The length of defect was 26.1 cm. **C** and **D** Anteroposterior (**C**) and lateral radiographs (**D**) showing the stability of the reconstruction system after 2 years. The bone growth could be observed proximally. **E** and **F** There was no limb-length discrepancy. The patient achieved full weight-bearing and was able to resume his original work. PMMA = polymethylmethacrylate

For shape-structural bone defects, because of the presence of a continuous bone cortex for lower extremity stabilization, the design of the prosthesis was fully compatible with the anatomical morphology of the defect area to ensure a complete fit to the host bone after implantation. Proximal and distal lateral plates were designed for fixation to ensure stable transmission of cortical stress from the defect site to the distal part, which provided a basis for early weight-bearing of the limb (Fig. 2). All patients did not require bone grafting.

### Postoperative management

The patients without osteoporosis or evidence of skin or soft tissue infection, were permitted to perform partial weight-bearing or knee flexion and extension training after removal of the drainage tube 1 week post-operatively. Patients with osteoporosis, skin flaps, or poor skin conditions were allowed to perform toe-touch weight-bearing with crutches 1 week post-operatively. Rehabilitation of full-range joint mobility training in bed was also encouraged, and full weight-bearing was gradually resumed within 2 months.

### Clinical and radiographic evaluation

Clinical and radiographic evaluations were conducted at 1, 3, and 6 months for the first year after surgery and every year thereafter. At each follow-up, a senior surgeon assessed the patients for infection relapse by probing the wound. The absence of sinus tract, material exposure, or pus within the wound was ensured. Infection eradication was characterized by a healed wound after one year of follow-up. Patients were asked to undergo full-length, frontal, and lateral radiographs of both lower extremities at each follow-up visit to observe prosthesis stability, proximal and distal bone cortical thickness, and bone growth. Union was considered to occur when the new bone grew into or climb over a porous structure. However, osseointegration was not observed upon radiographic examination; thus, we chose to observe trabecular bridging across the prosthesis-host junction on radiography and recorded the time of formation to indirectly evaluate the effect of osseointegration [32]. Based on evaluation of postoperative X-rays, union was defined if the following criteria were met: (1) prosthetic dislocation < 2 mm; (2) continuous bone formation across the bone interface and extent of prosthesis (proximal or distal); (3) no remaining infection; and (4) no periprosthetic translucent band on radiography. A dislocation of > 2 mm was defined as prosthetic loosening. Functional recovery was assessed using the lower extremity functional scale (LEFS). Neurovascular status, range of motion (ROM) of the knee joints, and any signs of infection were examined.

## Results

**Fig. 3:**
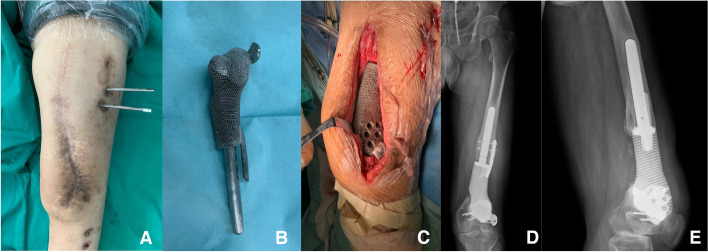
Anteroposterior and lateral radiographs at 36 months follow-up. **A** The wound healed well with no evidence of infection. **B** The metaphysis was considered insufficient to lock the intramedullary nail, an integrated intramedullary nail prosthesis was applied. **C** Prosthesis was stabilized using screws that penetrated the prosthesis. **D** Translucent zone surrounding the prothesis could be observed both on proximal and distal bone with no new bone growth. **E** Sclerosis of the distal interface

Demographic and follow-up data are detailed in Table 1 and laboratory data in Table 2. Among the eight patients with critical-size bone defects, five had metaphysis defects, one of whom used an integrated intramedullary nail prosthesis due to insufficient distal bone, and the others used a lateral plate-type prosthesis combined with an intramedullary nail. Three patients in whom the defect only involved the diaphysis were treated with a plain prosthesis and intramedullary nail. Among the three patients with shape-structural defects, two had involvement of the metaphysis, and one only had involvement of the diaphysis. Union was achieved in 10 (91%) patients according to our criteria. One patient who used an integrated intramedullary nail prosthesis was defined as having nonunion due to the presence of a translucent band on the distal bone after 36 months of follow-up, considering periprosthetic bone resorption (Fig. 3). The patient showed good functional performance at the latest follow-up visit. Among patients with shape-structural defects, one patient obtained fair results because of continuous postoperative pain with a visual analog scale (VAS) score of 4–5 and requirement for pain medication.

**Table 1:** Demographic data, infected data, details bone loss and treatment, and results of patients

Case	BMI (kg/m^2^)	Comorbidity	Types of bone defect	Sites and location (R/L)	Times of previous surgeries	Duration between 2 stages (d)
1	25.5	NA	Critical	R, Diaphysis	1	41
2	26.5	Stiff knee	Critical	R, Diaphysis	1	51
3	32.1	NA	Critical	L, Diaphysis	4	58
4^a^	21.3	Osteoporosis	Critical	L, Metaphysis	3	45
5	30.6	Stiff knee	Critical	R, Metaphysis	2	37
6	21.4	NA	Critical	L, Metaphysis	1	57
7	18.0	Osteoporosis	Critical	R, Metaphysis	2	49
8	27.0	NA	Critical	L, Metaphysis	3	59
9	25.6	Diabetes, Hypertension	Shape-structural	L, Diaphysis	2	68
10	27.4	NA	Shape-structural	R, Metaphysis	0	139
11	24.9	Hypertension, Venous thrombosis	Shape-structural	R, Metaphysis	0	66
Bone Loss (cm)	Times of debridement	Duration of Follow-up (mo)	External fixation time (d)	ROM	LEFS Score	Complications
12.0	6	68	93	0°–90°	60	Stiff knee
26.1	3	25	77	0°–40°	46	Stiff knee, Pin tract infection
10.4	4	24	99	0°–120°	61	Pin tract infection
12.5	5	61	186	0°–100°	53	Deep infection
11.5	4	56	66	0°–100°	62	-
8.5	3	51	56	0°–100°	49	Venous thrombosis
11.8	4	45	65	0°–110°	47	-
14.0	8	36	93	0°–90°	54	Pin tract infection
19.0	6	53	NA	0°–90°	52	-
17.8	2	30	NA	0°–120°	63	-
10.5	4	24	NA	0°–30°	41	Stiff knee, Postoperative Pain

Three patients developed postoperative stiffness; two of whom received early rehabilitation, and one of whom was subsequently managed with quadricepsplasty, and their ROM reached 90° at the latest follow-up. Of the latter two patients, one was still in the rehabilitation process with a knee ROM of 30°. ROM, range of motion; VSD, vacuum sealing drainage

*ROM* range of motion, *LEFS* lower extremity functional scale

^a^Deep infection occurred in one patient within 1 week after prosthesis implantation, which was treated with debridement, VSD, and intravenous antibiotic treatment. The patient was asked to stay in bed during treatment. After 1 month of treatment, the infection was successfully eradication, and removal of the prosthesis was avoided

**Table 2:** Infected data and laboratory examination results of patients

Case	Microorganism	WBC (^a^10^9/L)		C-rp (mg/L)		ESR (mm/h)	
		Stage 1^a^	Stage 2	Stage 1	Stage 2	Stage 1	Stage 2
1	Morganella morganii, Staphylococcus haemolyticus	7.02	8.67	2.13	1.01	44	11
2	Escherichia coli	4.82	7.78	1.82	0.80	37	20
3	Staphylococcus haemolyticus	6.21	6.65	4.56	0.52	45	12
4	Staphylococcus epidermidis, Klebsiella pneumoniae	4.35	5.38	1.75	1.18	25	10
5	Enterobacter cloacae	6.86	7.24	1.93	0.93	42	10
6	Negative culture	6.33	9.41	0.62	0.40	22	22
7	Staphylococcus haemolyticus	6.72	4.93	1.62	0.52	34	16
8	Staphylococcus aureus	8.85	8.50	1.96	0.63	27	11
9	Staphylococcus epidermidis	10.42	6.89	7.37	0.13	66	23
10	Staphylococcus aureus, Acinetobacter baumannii	11.73	8.16	2.62	1.14	43	3
11	Acinetobacter junii, enterococcus faecium	7.88	8.86	1.72	1.20	33	20

^a^Stage 1 referred to the laboratory examination results after the last debridement

### Radiological outcomes

In the critical-size defect group, two patients showed a prosthesis dislocation of < 2 mm compared with the immediate preoperative radiograph. However, no patient showed significant angulation or subsidence, and functional recovery was satisfactory. Proximal bone growth was observed in 6 patients at 6 months follow-up and in 1 patient at 12 months follow-up. Distal bone growth was observed in only two patients, and both bone defects only involved the diaphysis. One patient had an integral intramedullary nail, sclerosis of the distal interface, and a translucent zone surrounding the prosthesis, and no significant bone growth was observed until the last follow-up.

In patients with shape-structural defects, no dislocation was identified. Bone growth was observed at both the proximal and distal interfaces in 1 patient with diaphyseal defects at 12 months. In 2 patients with metaphysis, bone growth was observed unilaterally at 6 and 12 months, with significant osteosclerosis on the other side. Periprosthetic cortical thickening was observed in all patients, with no translucent bands Table 3.

**Table 3:** Radiological result

	Critical	Shape-structural
Dislocation	2	0
Interface osteosclerosis	1	2
Translucent band	1	0
Proximal bone growth	7	2
Distal bone growth	2	2

## Discussion

3D-printed protheses are most commonly used in the treatment of large bone defects after tumor resection to achieve rapid weight-bearing. However, in previous reports, the prosthesis design focused only on anatomical reconstruction with a solid stem. Aseptic loosening and periprosthetic fractures were the most common complication, with rates ranging 33–53.8% [10–14, 33], which limits the prothesis application. With technological developments, the novel porous structure design showed satisfactory osseointegration, providing long-term effectiveness of prostheses. Hence, we applied 3D-printed technology to treat infected bone defects to shorten the treatment cycle and compensate for graft bone insufficiencies. In our study, we reported a union rate of 91%, which was comparable to that of conventional treatment methods [1–8].

### 3D-printed porous prosthesis combined with an intramedullary nail fixation system improved performance and achieved reconstruction

To guarantee prosthesis stability and a controllable micromotion environment, we chose an intramedullary nail to disperse the stress. This effectively avoided peak stress being concentrated on the prosthesis, thereby reducing the risk of prosthetic or periprosthetic fracture. In diaphyseal defects, the load is transmitted from the proximal to the distal host bone to prevent stress-shielding effects and promote osteogenesis. In metaphyseal cases, because the plate was fixed with screws on the distal side, less stress was transmitted to the distal bone. The stress was concentrated on the lateral plate of the prosthesis, improving prosthesis stability and preventing the impact of rotation stress in the metaphysis.

Traditional customized prostheses are manufactured using one-piece structures. The stem was made into one piece with a prosthetic body and connected to a special piece. When used for femur reconstruction, the most frequent problems were aseptic stem loosening and structural failure, with rates ranging 16.7–57.0% [10, 12–14]. Benevenia [10] reported that 12 patients who underwent femur reconstruction with a prosthesis suffered implant-related complications, and a clamp-rod interface fracture was associated with cemented fixation of the stem. However, aseptic stem loosening was more likely to be caused by non-cemented fixation. Mahdal [13] also reported clamp-rod interface fractures after cement fixation, and the risk was higher when the defect length was > 10 cm [15] or involved meta-diaphyseal regions [14], indicating that stress was concentrated on the relatively unstable part of the prosthesis and not homogeneously distributed.

Porous prostheses seemingly have a lower implant-related complication rate than solid prostheses. Among 39 patients who received a custom 3D-printed implant, Abar [16] reported that 25.6% suffered implant loosening within 10 months. Zhao [11] used a porous prosthesis combined with a locking plate in 14 patients; none of whom developed implant-related complications at the final follow-up. Hammaa [34] used custom 3D-printed titanium truss cages combine with intramedullary nails also achieved favorable clinical outcomes with no occurrences of implant loosening. The formation of the bone bridge and the distance of infiltration into the implant may contribute to prosthesis stability.

According to our results, osseointegration mostly occurred at 6–12 months with no implant loosening or structural failure. In critical-sized defects, proximal osteogenesis started earlier than distal osteogenesis, and medial osteogenesis started earlier than lateral osteogenesis. However, owing to the stress-shielding effect, osteogenesis occurred later in distal metaphysis cases. Moreover, the prosthesis was fixed to the bone using intramedullary nailing, which limits the horizontal micromotion between the bone and prosthesis, enabling micromotion along the axis of the femoral stem, which differs from the traditional fixed system. As stability increased, micromotion disappeared, and bone growth halted [22]. Most patients in our study were allowed partial weight-bearing after drainage removal 1-week post-operatively, which may contribute to satisfactory functional outcomes and a shorter treatment period. However, dynamic stability and stress stimulation may enhance osseointegrative potential.

### Potential factors affecting osseointegration

Good osseointegration may improve prosthesis longevity, making it applicable for the treatment of non-tumor bone defects. In previous research [21, 22, 35], scaffolds with a porosity of 60–80% and pore sizes of 500-700um have been demonstrated to effectively promote bone ingrowth. The research [36] indicated that the scaffolds with a small pore size that smaller than 188um, the exchange of oxygen and nutrients would be affected, and failed to provide adequate surface area for cell adsorption and proliferation. Furthermore, by adjusting the porosity to match the elastic modulus of cortical bone (4 – 30 GPa) [37, 38], stress shielding could be prevented. Additionally, to avoid excessive weight of the implant, the entire implant also utilized a porous structure.

Bone formation within porous networks has previously been observed in bone tissue sections [22, 27, 28]; however, it cannot be directly observed in vivo. Therefore, in our study, we determined the bone ingrowth effect by observing periprosthetic bone formation and cortical thickness on radiography, which was reported previously [11, 16, 31]. We herein summarize several reasons to explain the different osteogenesis effects between proximal and distal bone: (1) enlarged sectional diameter of the distal femur dispersed stress; (2) the lower third of the femur has a poor degree of vascularization [39], which affects osteogenesis; and (3) the stress-shielding effect of the lateral plate of the metaphyseal prosthesis [22, 30], and the lack of micromotion due to rigid fixation. Therefore, we propose the necessity to conduct weight-bearing training within two weeks if feasible. In the shape-structural defect group, callus growth was found on the non-plate side and occurred slower than that in the critical defect group, which may be related to the locking screw fixation of the prosthesis.

At study onset, we hoped to retain more host bone to reduce surgical trauma and improve osseointegration; therefore, the bone interface did not flatten during the first stage. However, during long-term follow-up, in one patient with an integrated intramedullary nail prosthesis, the bone did not grow at the interface parallel to the lower limb force line. Bony sclerosis and transparent periprosthetic bands are observed at the bone interface, and resorption occurs at the contact interface because of the lack of stress stimulation. Therefore, we suggest the host bone interface be as flat as possible to ensure a uniform stress distribution and be perpendicular to the direction of the force line to ensure sufficient stress to stimulate new bone growth. Until the last follow-up, the patient had no significant decline in lower limb function, and we were not hurried to intervene.

The present study has several limitations. First, the sample size was small, and a larger sample size is needed to confirm the results. Second, the 3D-printed prosthesis was made of titanium alloy, which is not absorbable. Although the longest case achieved good treatment results more than 5 years after surgery, longer follow-up is needed to corroborate the effectiveness and safety of this treatment method. Third, there are no quantitative indicators to describe the osseointegration effect, and more cases are required to summarize the pattern of osteogenesis.

## Conclusion

Based on the current outcomes, the application of 3D-printed porous prosthesis could help to reconstruct local anatomy rapid and provided stable mechanical transmission. The application of the intramedullary nail provides mechanical dispersion and ensures the presence of micromotion, which can effectively shorten the treatment time, provide a relatively stable bone defect repair mode, avoid additional bone grafting, and achieve satisfactory clinical results in the medium-term; therefore, it is a potentially effective choice for the treatment of infected bone defects.

## Data Availability

Data is provided within the manuscript and supplementary information files. The datasets and the raw de-identified data used and/or analysed during the current study are available from the corresponding author on reasonable request.

## Abbreviations

3D: Three-dimensional
PMMA: Polymethylmethacrylate
VSD: Vacuum sealing drainage
WBC: White blood cell
C-rp: C-reactive protein
ESR: Erythrocyte sedimentation
CT: Computed tomography
LEFS: Lower extremity functional scale
ROM: Range of motion
VAS: Visual analog scale

## References

[1] Jayaramaraju D, Venkataramani H, Rajasekaran RB (2019). Modified Capanna’s Technique (Vascularized Free Fibula Combined with Allograft) as a single-stage procedure in post-traumatic long-segment defects of the lower end of the femur: outcome analysis of a series of 19 patients with an average gap of 14 cm. Indian J Plast Surg.

[2] Baud A, Flecher X, Rochwerger RA (2020). Comparing the outcomes of the induced membrane technique between the tibia and femur: retrospective single-center study of 33 patients. Orthop Traumatol Surg Res.

[3] Masquelet AC (2020). The induced membrane technique. Orthop Traumatol Surg Res.

[4] Masquelet A, Kanakaris NK, Obert L (2019). Bone repair using the Masquelet technique. J Bone Joint Surg Am.

[5] Salcedo Cánovas C, Martínez Ros J, Ondoño Navarro A (2021). Infected bone defects in the lower limb management by means of a two-stage distraction osteogenesis protocol. Eur J Orthop Surg Traumatol.

[6] Borzunov DY, Kolchin SN (2022). Nonunion of the femoral shaft associated with limb shortening treated with a combined technique of external fixation over an intramedullary nail versus the Ilizarov method. Arch Orthop Trauma Surg.

[7] Bas A, Daldal F, Eralp L (2020). Treatment of tibial and femoral bone defects with bone transport over an intramedullary nail. J Orthop Trauma.

[8] Dheenadhayalan J, Devendra A, Velmurugesan P (2022). Reconstruction of massive segmental distal femoral metaphyseal bone defects after open injury: a study of 20 patients managed with intercalary gamma-irradiated structural allografts and autologous cancellous grafts. J Bone Joint Surg Am.

[9] Cuvillier M, Meucci JF, Cazorla C (2022). Masquelet’s induced membrane technique associated with Reamer Irrigation Aspiration grafting and intramedullary Nailing (MaRIAN) for chronic diaphyseal osteomyelitis of the lower limb. Orthop Traumatol Surg Res.

[10] Benevenia J, Kirchner R, Patterson F (2016). Outcomes of a modular intercalary endoprosthesis as treatment for segmental defects of the femur, tibia, and humerus. Clin Orthop Relat Res.

[11] Zhang Z, Shi Y, Fu J (2022). Customized three dimensional printed prosthesis as a novel intercalary reconstruction for resection of extremity bone tumours: a retrospective cohort study. Int Orthop.

[12] Lun DX, Hu YC, Yang XG (2018). Short-term outcomes of reconstruction subsequent to intercalary resection of femoral diaphyseal metastatic tumor with pathological fracture: comparison between segmental allograft and intercalary prosthesis. Oncol Lett.

[13] Mahdal M, Pazourek L, Apostolopoulos V (2022). Outcomes of Intercalary endoprostheses as a treatment for metastases in the femoral and humeral diaphysis. Curr Oncol.

[14] Streitbürger A, Hardes J, Nottrott M (2022). Reconstruction survival of segmental megaendoprostheses: a retrospective analysis of 28 patients treated for intercalary bone defects after musculoskeletal tumor resections. Arch Orthop Trauma Surg.

[15] Ruggieri P, Mavrogenis AF, Bianchi G (2011). Outcome of the intramedullary diaphyseal segmental defect fixation system for bone tumors. J Surg Oncol.

[16] Abar B, Kwon N, Allen NB (2022). Outcomes of surgical reconstruction using custom 3D-printed porous titanium implants for critical-sized bone defects of the foot and ankle. Foot Ankle Int.

[17] Tetsworth K, Woloszyk A, Glatt V (2019). 3D-printed titanium cages combined with the Masquelet technique for the reconstruction of segmental femoral defects: preliminary clinical results and molecular analysis of the biological activity of human-induced membranes. OTA Int.

[18] Tetsworth K, Block S, Glatt V (2017). Putting 3D modelling and 3D printing into practice: virtual surgery and preoperative planning to reconstruct complex post-traumatic skeletal deformities and defects. SICOT J.

[19] Lu Y, Chen G, Long Z (2019). Novel 3D-printed prosthetic composite for reconstruction of massive bone defects in lower extremities after malignant tumor resection. J Bone Oncol.

[20] Chen Y, Frith JE, Dehghan-Manshadi A (2017). Mechanical properties and biocompatibility of porous titanium scaffolds for bone tissue engineering. J Mech Behav Biomed Mater.

[21] Chen Z, Yan X, Yin S (2020). Influence of the pore size and porosity of selective laser melted Ti6Al4V ELI porous scaffold on cell proliferation, osteogenesis and bone ingrowth. Mater Sci Eng C Mater Biol Appl.

[22] Zhang T, Wei Q, Zhou H (2021). Three-dimensional-printed individualized porous implants: a new “implant-bone” interface fusion concept for large bone defect treatment. Bioact Mater.

[23] Ji T, Yang Y, Tang X (2020). 3D-printed modular hemipelvic endoprosthetic reconstruction following periacetabular tumor resection: early results of 80 consecutive cases. J Bone Joint Surg Am.

[24] Liu W, Shao Z, Rai S (2020). Three-dimensional-printed intercalary prosthesis for the reconstruction of large bone defect after joint-preserving tumor resection. J Surg Oncol.

[25] Wang J, An J, Lu M (2021). Is three-dimensional-printed custom-made ultra-short stem with a porous structure an acceptable reconstructive alternative in peri-knee metaphysis for the tumorous bone defect?. World J Surg Oncol.

[26] Zhang Y, Lu M, Min L (2021). Three-dimensional-printed porous implant combined with autograft reconstruction for giant cell tumor in proximal tibia. J Orthop Surg Res.

[27] Hou G, Liu B, Tian Y (2020). An innovative strategy to treat large metaphyseal segmental femoral bone defect using customized design and 3D-printed micro-porous prosthesis: a prospective clinical study. J Mater Sci Mater Med.

[28] Hou G, Liu B, Tian Y (2022). Reconstruction of ipsilateral femoral and tibial bone defect by 3D-printed porous scaffold without bone graft: a case report. JBJS Case Connect.

[29] Safali S, Berk T, Makelov B (2023). The possibilities of personalized 3D printed implants-a case series study. Medicina (Kaunas).

[30] Liu B, Li X, Qiu W (2022). Mechanical distribution and new bone regeneration after implanting 3D-printed prostheses for repairing metaphyseal bone defects: a finite element analysis and prospective clinical study. Front Bioeng Biotechnol.

[31] Burger D, Pumberger M, Fuchs B (2016). An uncemented spreading stem for the fixation in the metaphyseal femur: a preliminary report. Sarcoma.

[32] Nwankwo EC, Chen F, Nettles DL (2019). Five-year follow-up of distal tibia bone and foot and ankle trauma treated with a 3D-printed titanium cage. Case Rep Orthop.

[33] Errani C, Tsukamoto S, Almunhaisen N (2021). Intercalary reconstruction following resection of diaphyseal bone tumors: a systematic review. J Clin Orthop Trauma.

[34] Gamieldien H, Ferreira N, Birkholtz FF (2023). Filling the gap: a series of 3D-printed titanium truss cages for the management of large, lower limb bone defects in a developing country setting. Eur J Orthop Surg Traumatol..

[35] Lv J, Xiu P, Tan J (2015). Enhanced angiogenesis and osteogenesis in critical bone defects by the controlled release of BMP-2 and VEGF: implantation of electron beam melting-fabricated porous Ti6Al4V scaffolds incorporating growth factor-doped fibrin glue. Biomed Mater.

[36] Chang B, Song W, Han T (2016). Influence of pore size of porous titanium fabricated by vacuum diffusion bonding of titanium meshes on cell penetration and bone ingrowth. Acta Biomater.

[37] Yan X, Li Q (2019). Mechanical and in vitro study of an isotropic Ti6Al4V lattice structure fabricated using selective laser melting. J Alloys Compounds.

[38] Funk JR, Kerrigan JR, Crandall JR (2004). Dynamic bending tolerance and elastic-plastic material properties of the human femur. Annu Proc Assoc Adv Automot Med.

[39] Santolini E, Goumenos SD, Giannoudi M (2014). Femoral and tibial blood supply: a trigger for non-union?. Injury.

